# Mathematical evaluation of jumping distance in total hip arthroplasty: Influence of abduction angle, femoral head offset, and head diameter

**DOI:** 10.3109/17453670902988378

**Published:** 2009-06-01

**Authors:** Elhadi Sariali, Jean Yves Lazennec, Frederic Khiami, Yves Catonné

**Affiliations:** ^1^Hôpital Pitié SalpétrièreParisFrance; ^2^Institute of Medical and Biological Engineering, University of LeedsLeedsUK

## Abstract

**Background and purpose** The jumping distance (JD) is the degree of lateral translation of the femoral head center required before dislocation occurs. The smaller the distance, the higher the theoretical risk of dislocation. The aim of our study was to evaluate this jumping distance and its variation according to the characteristics of the implant, and also the theoretical gain in using large head diameters of above 38 mm.

**Methods** The JD was calculated as a function of the cup ante-version and abduction angles, the head diameter, and the head offset (defined as the distance between the center of the femoral head and the cup opening plane). Head diameters of 28, 32, 36, 40, 44 and 48 mm were analyzed. The abduction angle was increased from 0° to 80° with a 10° increment. The anteversion angle was increased from 0° to 40° with a 5° increment.

**Results** The jumping distance was found to decrease as the cup abduction angle increased (0.25 mm each 1° for 32-mm head diameter). It increased by 0.05 mm for a 1° increase in the ante-version angle. The jumping distance increased as the head diameter increased (0.4 mm each mm diameter for a 45° abduction angle). The net gain obtained by increasing the diameter, however, decreased when abduction angle increased (0.25 each mm diameter for 60° abduction). The JD decreased by 0.92 mm for each 1-mm increase in head offset, showing that head offset was the most important parameter influencing the JD.

**Interpretation** The theoretical gain in stability obtained by using a large femoral head (above 36 mm) is negligible in cases where there is a high cup abduction angle. An increase in offset of the femoral head substantially reduces the jumping distance and it should therefore be avoided.

## Introduction

Dislocation rates of between 0.5% and 10% are reported for primary THA (Sariali et al 2008), and these increase to between 10% and 25% after revision surgery (Alberton et al 2002). Dislocation remains the second most common reason for revision surgery, after aseptic loosening. Many factors influence the dislocation risk and these factors can be classified as three main types: patient characteristics, the surgical technique, and the prosthesis design. Abductor muscle efficiency (Kung and Riers 2007), patient cooperativity, and neurological disease (Sariali et al. 2008) are important influencing factors. Concerning the surgical technique, the restoration of hip anatomy—including center of hip rotation, the femoral offset, lower limb length (Malik et al. 2007), and correct anteversion angles (Jolles et al. 2002)—are crucial for hip stability. For the prosthesis design, head-to-neck ratio, head diameter, and femoral head offset are known to influence the risk of dislocation (Scifert et al. 2001).

**Figure 1.**
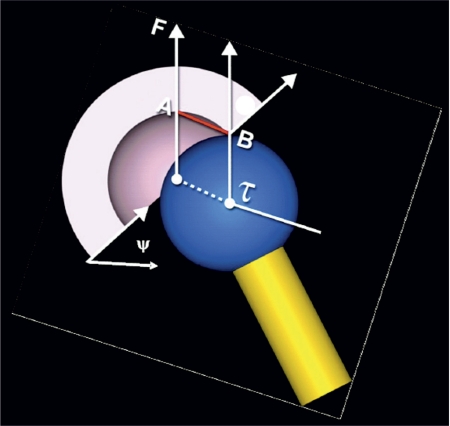
The jumping distance is the lateral translation (AB) of the center of the femoral head (t) before dislocation occurs. F is the load force and y is the planar cup inclination angle measured in the frontal plane.

Large head diameter is an attractive option to reduce the dislocation risk after total hip replacement. In fact, Scifert et al. (2001) showed an increase in resisting moment to dislocation of 3.6% per mm increase in head diameter. However, the literature on the use of large head diameters in revision surgery (Beaule et al. 2002, Amstutz et al. 2004, Fricka et al. 2006, Kung and Riers 2007) still shows a failure rate of between 8% and 40%, with no statistically significant difference between standard femoral head sizes (28, 32 mm) and large ones (Kung and Riers 2007). Some authors have proposed the use of jumping distance as a predictive factor for dislocation (Lazennec et al. 2006). The jumping distance is the degree of lateral translation of the femoral head center required for dislocation to occur (Figure 1). The lower the jumping distance, the higher the theoretical risk of dislocation. We evaluated this jumping distance and its variation according to implant characteristics—in particular, the cup abduction, the femoral head diameter, and the head offset, in order to determine the net theoretical gain when using large femoral heads.

## Material and methods

### Definition of the parameters

**Figure 2.**
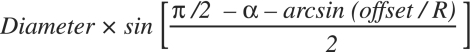
Definition of the angular parameters used for positioning of the cup. O is the center of the cup, Oz is the cranio-caudal axis, Oy is the lateral-medial axis, and Ox is the postero-anterior axis. Starting at the reference position (I), a first rotation of value a (cup abduction angle) is performed around the anterior-posterior axis (II). A second rotation of value b (cup anteversion angle) is performed around the cranialcaudal axis (III).

A Cartesian reference landmark was defined: O was the center of the cup, Oz was the cranio-caudal axis, Oy was the lateral-medial axis, and Ox the postero-anterior axis (Figure 2). The cup was positioned by performing two rotations. Firstly, we made a rotation around the Ox axis of a value α, which was called the cup frontal abduction angle. Secondly, we made a rotation around Oz, of a value β that corresponded to the cup anteversion angle as defined by Murray (1993). The planar cup inclination angle (Ψ) measured on X-rays, can be calculated according to the cup frontal abduction angle and the cup anteversion angle using formula 1 (Appendix 1):

**Figure:**
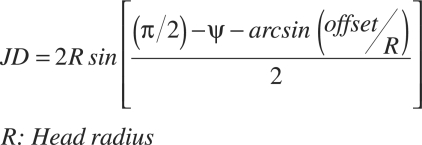


**Figure 3.**
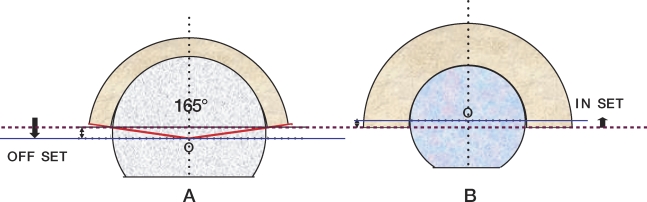
The femoral head offset is the distance from the center of the head (O) to the opening plane of the cup (purple line). If the head center is outside the cup, the offset is positive (A); otherwise it is negative and is called inset (B). The use of large heads above 38 mm in diameter generally imposes the use of an offset because the cup is usually a truncated hemisphere of 165° for the large heads.

The offset of the femoral head (offset) was defined as the distance between the femoral head center and the cup opening plane (Figure 3). If the femoral center was located inside the cup, the offset was negative and the absolute value was named the femoral inset, whereas if it was situated outside the cup the offset was positive.

The jumping distance can be calculated using formula (2) (Appendix 2):

**Figure:**
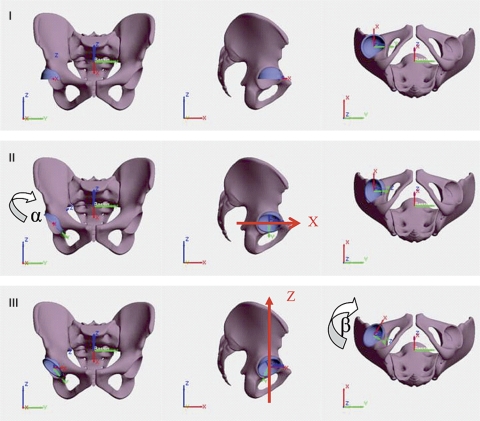


### Method

We examined femoral head diameters of 28, 32, 36, 40, 44, and 48 mm. The acetabular abduction varied from 20° to 80° with a 5° increment. The acetabular anteversion varied from 0° to 40° with an increment of 5°. The value of the head offset varied from –2 mm inset to +5 mm offset. In fact, head diameter and head offset are not independent parameters. The large head diameters currently available (above 38 mm) are generally used with cups corresponding to a truncated hemisphere of about 165°. This design generates an offset that increases as the diameter increases (Figure 3). On the the other hand, small head diameters below 32 mm have an inset.

We analyzed the variation in jumping distance according to the abduction angle, the acetabular cup anteversion, the head diameter, and the femoral head offset. The gain in JD was determined for each mm or degree of variation of these parameters. Finally, we used current commercial designs of small and large heads in order to determine the variation in the JD according to the head diameter. For the 28-, 32-, and 36-mm diameters, the data for the offset were those of ceramic-on-ceramic THR and they were provided by Ceram-Tec (Plochingen, Germany). The heads of 28-mm and 32-mm diameter have an inset of 1 mm, whereas the heads of 36-mm diameter have an offset of 0 mm. For the large sizes (40, 44, and 48 mm), the data used for the offset were those of the DUROM implants as provided by Zimmer (Warsaw, IN).

## Results

### Variation according to cup abduction and anteversion angles

**Figure 4.**
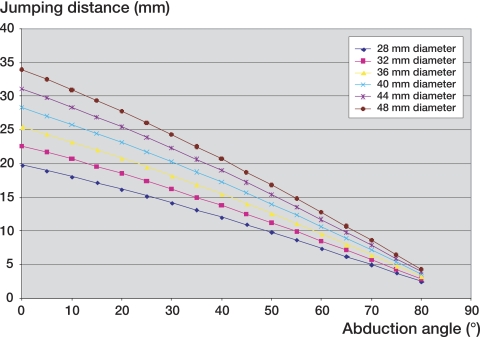
Variation in jumping distance according to the cup abduction angle

**Figure 5.**
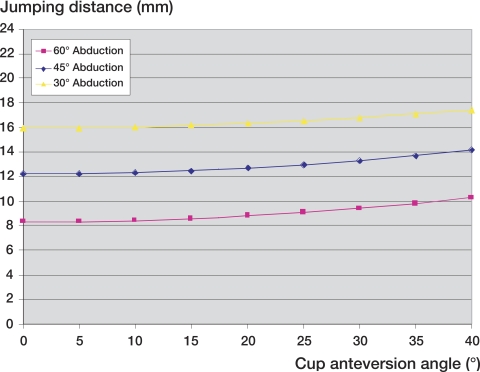
Variation in jumping distance according to acetabular ante-version angle.

The jumping distance decreased as the abduction angle increased (Figure 4). The decrease was about 0.25 mm for each 1° of increase in abduction angle. Thus, when using a 32-mm head diameter, an increase of 10° in the abduction angle induced a decrease of about 2.5 mm (20%) in the jumping distance. On the other hand, JD increased as the acetabular anteversion increased (Figure 5), but this variation was much lower than the variation according to abduction angle. In fact, when using a 32-mm head diameter, an increase of 10° in cup anteversion angle induced an increase of 0.5 mm in the jumping distance (4%). Thus, the anteversion effect could be disregarded and the formula for the jumping distance could be simplified using only the abduction angle α.

### Variation according to head diameter

**Figure 6.**
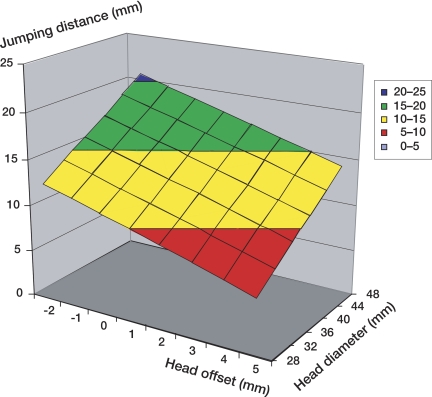
Combined influence of head offset and diameter on the jumping distance. 45° abduction and 15° anteversion cup angles are used.

The jumping distance increased as the femoral head diameter increased (Figure 6). However, this gain became substantially reduced as the abduction angle increased; in fact, the gain in JD for each mm increase in head diameter was about 0.5 mm/ mm for 30° abduction angle, 0.4 mm/mm for 45° abduction angle, and 0.25 mm/mm for 60° abduction angle. The gain in JD was therefore minimal for high abduction angles.

### Variation according to head offset

**Figure 7.**
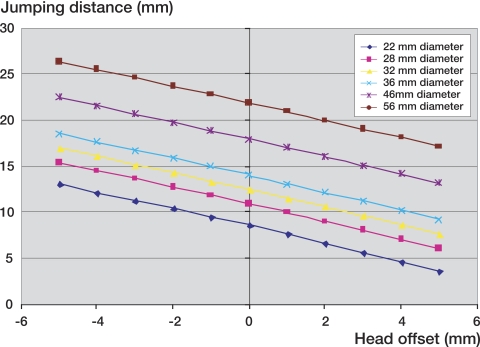
Influence of femoral offset on jumping distance.

The head offset was the geometrical factor that has the highest influence on the jumping distance. In fact, a decrease in JD of 0.92 mm was found for a 1-mm increase in head offset (Figure 7). When using large head diameters, given that the head offset is generally increased by about 3 mm, the jumping distance is 2.76 mm lower than the theoretical value corresponding to a complete spherical cup design.

### Variation according to head diameter for 2 current commercial designs

**Figure 8.**
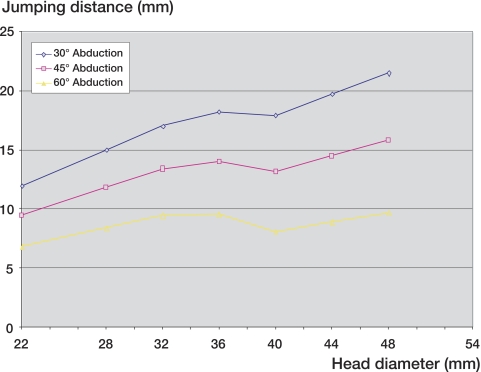
Dependency of jumping distance on head diameter for current commercial designs. For diameters above 38 mm, the cup is an incomplete truncated hemisphere, causing a positive offset, which in turn causes a dip in the jumping distance.

The jumping distance increased as the femoral head diameter increased, except between 36 and 40 mm where a decrease of 1 mm (6%) was found (Figure 8). Thus, the JD was similar for 32-mm and 40-mm head diameter. For an abduction angle of 45°, JD was 14.1 mm for a 36-mm head diameter and 15.8 mm for a 48-mm diameter, giving an increase of 1.7 mm (12%). However, this gain decreased as the abduction angle increased; indeed, for an abduction angle of 60°, the difference in jumping distance between a 36-mm diameter head and a 48 mm-diameter head was minimal: about 0.13 mm (1.4%)

## Discussion

We have not found any studies in the literature on the jumping distance and its variations according to the cup abduction angle, the cup anteversion angle, and the head offset. For a given head diameter, this lateral distance decreases as the abduction angle increases, making the risk of dislocation theoretically higher. Bader and Willmann (1999) reported similar results using a 3-dimensional CAD simulation, confirming that if the cup is too vertical, the risk of dislocation increases because of impingement between the femoral neck and the rim. This appears to be confirmed also by the clinical results reported by Kummer et al. (1999) who found that if cup inclination was superior to 45°, there was a substantial limitation in internal and external rotation, at the same time increasing the risk of impingement and dislocation.

Jumping distance was found to decrease as the head offset increased. This parameter is the more important factor, as it generates higher variability in JD. Our results are similar to those in the literature. Indeed, Scifert et al. (2001) found an increasing stability by the inset of the center of rotation. They reported that the resisting moment to dislocation increased by 5.8% for each 1 mm of inset. Thus, increase in head offset reduces the jumping distance, and theoretically it should be avoided as far as possible.

For large head diameters (above 38 mm), there is frequently a head offset of about 3 mm because the cup design corresponds to a truncated hemisphere of 165°, in order to minimize the bone removal during the reaming procedure, and to increase the theoretical range of motion. This is why the use of large head diameters leads to less important jumping distances than those expected. This may explain the similar dislocation rates for heads of 32-mm diameter and for larger heads (Beaule et al. 2002, Amstutz et al. 2004, Fricka et al. 2006).

When we used offset values of designs that are currently available, the jumping distance increased as the head diameter increased, except between 36 and 40 mm, where a mean decrease of 1 mm (6%) occurred. Scifert et al. (2001) reported increased stability with large head diameter, but did not analyze heads with diameters of above 32 mm, which often include an offset. Furthermore, as the abduction angle increased, the advantage gained from the large diameter disappeared. In fact, no difference was found between a 36-mm and a 48-mm head diameter for a 60° abduction angle. With a high abduction angle such as 60°, the JD is lower for 40-mm or 44-mm diameter than for 36-mm diameter. This shows that when the cup is too vertical, the theoretical risk of dislocation is higher when using a 40-mm or a 44-mm head than with a 36-mm head. This is due to the fact that when using head diameters above 36 mm, the head offset generally increases by about 3 mm, thus leading to a decrease in the jumping distance.

Dislocation after THR is nevertheless a multifactorial phenomenon and cannot be analyzed with only the jumping distance as a predictive parameter. Soft tissue balance seems to be a key point for hip stability after THR. Indeed, Kung and Riers (2007) reported no difference between large heads and small heads regarding stability when there was no abductor mechanism.

Although the use of large femoral heads (above 38 mm) is an attractive option for prevention of dislocation, it may not solve the problem of instability after THR revision. The increase in head offset reduces the jumping distance substantially and should therefore be avoided as much as possible.

## Appendix 1

The frontal cup inclination angle (Ψ) corresponds to the projection of the abduction angle (α) on the frontal plane. These 2 angles are different because of the anteversion angle (β), which corresponds to a rotation around the axis (oZ). This planar cup inclination is calculated on pelvic X-rays in clinical practice. N is the vector perpendicular to the cup opening plane. T is a vector directed along the intersection of the frontal plane and the cup opening plane (T is the long axis of the ellipse usually seen on the anterior-posterior radiographs).

## Appendix 2

Calculation of the jumping distance according to the frontal cup inclination angle (Ψ), the anteversion angle (β), the radius of the head (R) and the head offset.
